# Primary disease sites and patterns of spread in cases of neurolymphomatosis in the orbit associated with lymphoma

**DOI:** 10.1186/s40644-021-00409-3

**Published:** 2021-05-26

**Authors:** Seth J. Fritzhand, Bita Esmaeli, Jia Sun, J. Matthew Debnam

**Affiliations:** 1 School of Nursing and Health Studies, Georgetown University, Washington, DC, USA; 2 Orbital Oncology & Ophthalmic Plastic Surgery, Department of Plastic Surgery, The University of Texas MD Anderson Cancer Center, Houston, TX USA; 3 Department of Biostatistics, The University of Texas MD Anderson Cancer Center, Houston, TX USA; 4 Department of Neuroradiology, The University of Texas MD Anderson Cancer Center, 1400 Pressler Blvd., Unit 1482, Houston, TX 77030 USA

**Keywords:** Neurolymphomatosis, Lymphoma, Cranial nerves, Orbits

## Abstract

**Background:**

Neurolymphomatosis involving the cranial nerves (CNs) is rare. We sought a better understanding of the primary disease sites and patterns of spread in neurolymphomatosis of the orbit and retro-orbital cranial nerves.

**Methods:**

Patients with lymphoma and MRI evidence of neurolymphomatosis of CN II, III, IV, V_1_, or V_2_ were retrospectively reviewed. Demographics and primary disease site and sites of neurolymphomatosis on MRI were recorded. Wilcoxon rank sum test was used to compare number of sites of neurolymphomatosis with lymphoma type and survival.

**Results:**

The study included 18 patients. The most frequent types of lymphoma were diffuse large B-cell (DLBCL) (*n* = 9) and marginal zone (*n* = 3). In 9 patients, lymphoma presented as a mass (*n* = 7) or infiltrative disease (*n* = 2) directly involving the orbit; in 6, a maxillofacial mass spread directly to CNs; and in 3, lymphoma at remote sites spread to orbital CNs. Overall, 81 sites of neurolymphomatosis were noted. The most common sites were the maxillary nerve (V_2_) including at the infraorbital fissure or foramen rotundum (17 patients; 19 nerves), pterygopalatine fossa (16 patients; 19 nerves), and cavernous sinus (9 patients; 12 nerves). Number of sites of neurolymphomatosis was significantly lower for DLBCL than for other lymphoma types (*p* = 0.007). Number of sites of neurolymphomatosis did not affect survival (*p* = 0.26). The mean interval between the pathologic diagnosis and MRI documentation of the full extent of neurolymphomatosis was 39 days after pathologic diagnosis.

**Conclusions:**

Based on our study results, neurolymphomatosis in the orbit appears to be frequently associated with an orbital and/or maxillofacial mass and commonly involves CN V_2_, the pterygopalatine fossa, and the cavernous sinus. DLBCL may be associated with fewer sites of neurolymphomatosis than other lymphomas. In patients with lymphoma, a systematic search for neurolymphomatosis is imperative for early detection.

## Background

 Non-Hodgkin Lymphomas (NHLs) are a class of lymphoproliferative malignancies that may arise in extra-nodal locations and often involve both nodal and extra-nodal sites [1]. New treatments for NHL are leading to longer survival times and lower mortality, and this may increase the incidence of central nervous system disease involvement. Thus, the number of patients presenting with ophthalmologic sequelae of NHL may increase [2].

Different types of malignancies can directly involve the cranial nerves which is termed perineural spread (PNS). When specific to hematological malignancies, including lymphoma, this spread along the cranial and peripheral nerves is termed neurolymphomatosis, which is rare. PNS and neurolymphomatosis are different from leptomeningeal disease (LMD) which is the spread of a malignancy to the cerebrospinal fluid with involvement of the leptomeninges including the arachnoid, subarachnoid space, and pia mater, and also includes the cranial nerves. Neurolymphomatosis affects patients with NHL and lack of detection may lead to a delay in diagnosis [3, 4]. The incidence of neurolymphomatosis is increasing worldwide, and this may be due to increased awareness and earlier detection with more sophisticated diagnostic techniques [5].

To the best of our knowledge, there are no large case series and only a few reports in the literature about neurolymphomatosis occurring in patients with lymphoma in or near the orbit [2, 6–8]. To improve understanding of neurolymphomatosis in the orbit and retro-orbital cranial nerves associated with NHL, we reviewed the demographics and MR imaging findings of patients with this presentation who were treated at our institution, a major cancer center.

## Methods

This retrospective study was approved by the Institutional Review Board at The University of Texas MD Anderson Cancer Center. The research was HIPAA compliant and adhered to the ethical principles in the Declaration of Helsinki as amended in 2013. Our radiological database was searched to identify patients, both children and adults, who received a pathologic diagnosis of lymphoma at our institution between August 2005 and June 2019 and had magnetic resonance imaging (MRI) evidence of neurolymphomatosis involving the CNs closest to the orbit, specifically CN II, III, IV, V_1_, or V_2_. A total of 18 patients were identified who met these criteria, and these patients were included in the study.

A head and neck radiologist (JMD) with over 20 years of experience reviewed each of the MRI studies for evidence of neurolymphomatosis, determined the extent of disease, and recorded the presence of any enhancement representing NL in CN II through CN XII, the pterygopalatine fossa (PPF), the vidian canal, and Meckel’s cave. Neurolymphomatosis involving the superior orbital fissure was included as part of CN V_1_, and neurolymphomatosis involving the infraorbital fissure or foramen rotundum was classified together with neurolymphomatosis involving CN V_2_. The reviewed MR images of the orbits included axial T1 pre-contrast without fat saturation, orthogonal T1 post-contrast with fat saturation and axial T2 with fat saturation.

Data about survival were also obtained. The Wilcoxon rank sum test was used to correlate the number of sites of neurolymphomatosis with disease type and survival.

## Results

The 18 patients in the study ranged in age from 24 to 78 years (mean, median [SD] 61, 63 [13] years) (Table 1). Lymphoma was diagnosed by biopsy in 17 patients and by lumbar puncture in 1. The type of lymphoma was DLBCL in 9 patients, marginal zone lymphoma in 3, other forms of low-grade B-cell lymphoma in 2, mantle cell lymphoma in 2, NK/T-cell lymphoma in 1, and chronic lymphocytic leukemia/small lymphocytic lymphoma in 1 patient. MRI documentation of neurolymphomatosis preceded pathologic diagnosis of neurolymphomatosis (by up to 59 days) in 5 patients and followed pathologic diagnosis of neurolymphomatosis (by up to 176 days) in 13 patients; the mean interval (SD) between the pathologic diagnosis and MRI documentation was 39 (62) days after pathologic diagnosis. In 8 patients the lymphoma was recurrent; in these patients, the interval between initial diagnosis and diagnosis of recurrence ranged from 6 months to 11 years (mean [SD], 4.6 [4.7] years).

**Table 1:** Demographic and clinical characteristics in 18 patients with neurolymphomatosis in the orbit associated with lymphoma

Patient *#*	Age,y/ sex	Lymphoma subtype	Prior disease sites	Time to recurrence	Symptoms	Treatment
1	64/M	DLBCL	NA	NA	maxillary sinus mass with orbital extension, periorbital edema and epiphora	CHOP/Bleo followed by interferon
2	72/F	DLBCL	Skull base	10 years	left cheek mass with cranial neuropathies and diplopia	R-ESHAP, R-CHOP
3	70/F	DLBCL	Uterus	1.5 years	facial palsy and trigeminal neuralgia	R-CHOP radiation (30 Gy)
4	73/M	DLBCL	NA	NA	maxillary sinus/cheek mass with infraorbital nerve PET/CT activity	R-CHOP, stem cell transplant
5	51/M	DLBCL	NA	NA	lacrimal gland/orbital mass with optic disc edema and optic neuropathy	R-DHAP
6	59/M	DLBCL	NA	NA	diplopia/maxillary sinus mass with secondary orbital involvement	R-CHOP
7	60/M	DLBCL	NA	NA	presented with nasopharyngeal mass with secondary orbital involvement; also diplopia due to multiple cranial neuropathies including 3rd, 4th and 6th nerves	R-CHOP
8	51/M	DLBCL	Eyebrow	6 months	presented with an eyebrow mass (which was lymphoma on biopsy) as well as a maxillary sinus mass with V1 infiltration on MRI	R-CHOP, methotrexate
9	75/M	DLBCL	Head & neck nodes	2 years	presented with cheek mass with V2 and pterygopalatine involvement	R-EPOCH, methotrexate
10	58/F	Marginal zone	Chest	11 years	proptosis	Rituxan-hyper CVAD
11	62/M	Marginal zone	Chest, abdomen	1 year	presented with an asymptomatic orbital/pterygoplatine infiltrative mass that was lymphoma; otherwise asymptomatic	ultra low dose XRT (4 Gy)
12	24/M	Marginal zone	NA	NA	Long standing visual loss and optic neuropathy also with orbital and pterygopalatine mass with intracranial extension biopsy proven to be lymphoma	R-EPOCH, methotrexate
13	54/M	Low-grade B-cell	NA	NA	presented with a posterior orbital mass; no specific ocular or periocular symptoms	Ibrutinib, Rituxan and Temodar
14	72/F	Low-grade B-cell	NA	NA	proptosis/orbital mass	SMILE with IT chemotherapy
15	69/M	Mantle cell	NA	NA	presented with upper eyelid and periorbital lesions; MRI showed bilateral orbital masses	R-CHOP
16	48/F	Mantle cell	Ovary	9 months	facial and jaw pain and parasthesias on the cheek	R CHOP
17	78/M	CLL/SLL	Abdomen	10 years	third nerve palsy /diplopia	R-ICE
18	66/M	NK/T-cell	NA	NA	presented with a nasal cavity mass with involvement of the nasolacrimal duct and secondary epiphora (tearing)	R-HyperCVAD/ R-MTX/ R-MA

^a^NA indicates that disease was primary disease

*CLL/SLL* chronic lymphocytic leukemia/small lymphocytic lymphoma, *DLBCL* diffuse large B-cell lymphoma, *NK* natural killer

### Primary disease sites and sites of neurolymphomatosis

**Fig. 1:**
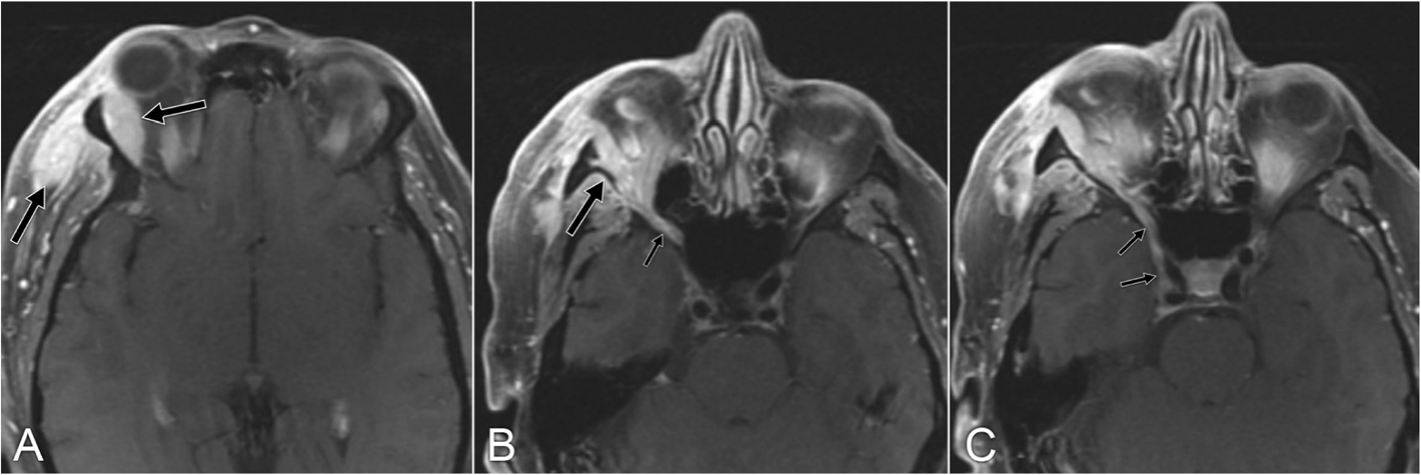
Axial postcontrast MR images of a 51-year-old-man with DLBCL who presented with diplopia (patient 5). **a** Mass in the right lacrimal gland and masticator space (arrows). **b** Extension from the orbital mass (large arrow) through the inferior orbit fissure (small arrow) into the PPF. **c** Involvement of the foramen rotundum and cavernous sinus (arrows)

**Fig. 2:**
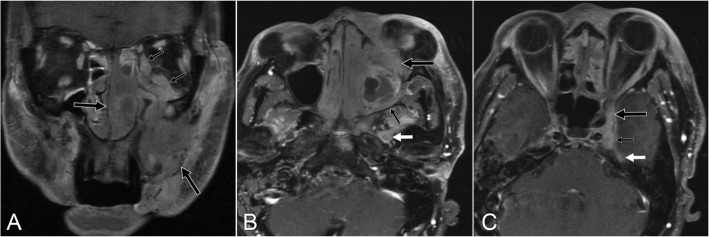
Postcontrast MR images of a 66-year-old-man with NK/T-cell lymphoma who presented with facial numbness (patient 18). **a** Coronal image shows a mass in the left side of the face and nasal cavity (large arrows) with infiltration of the orbit including the extra-ocular muscles (small arrows). **b** Axial image shows the orbital mass (large black arrow) and extension through the inferior orbit fissure to the pterygopalatine fossa (small black arrow). Involvement of the mandibular nerve (V3) is also present (white arrow). **c** Axial images shows involvement of the cavernous sinus (large black arrow) and Meckel’s cave (small black arrow). There is spread via the superficial petrosal nerve (white arrow) to the facial nerve (not shown)

**Fig. 3:**
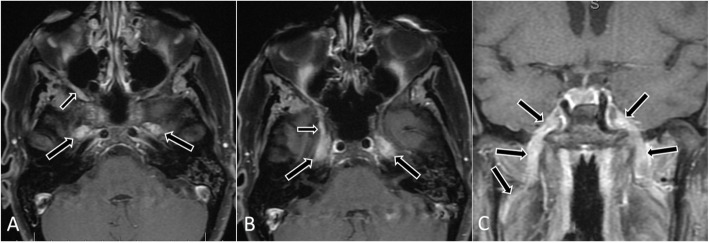
Postcontrast MR images of a 58-year-old-woman with relapsed Mantle cell lymphoma who presented with facial and jaw pain and parasthesias on the cheek from hematogenous spread (patient 16). **a** Axial image demonstrates involvement of bilateral mandibular nerves (V3) (large arrows) and the right maxillary nerve (V2) (small arrow). **b** Axial image demonstrates lymphoma in bilateral Meckle’s caves (large arrows) and the right foramen rotundum (V2) (small arrow). **c** Coronal images demonstrating involvement of bilateral mandibular nerves (V3) (arrows)

In 9 patients, lymphoma directly involved the orbit in the form of a mass (*n* = 7) (Fig. 1) or infiltrative disease (*n* = 2) (Table 2). In 6 patients, a maxillofacial mass spread directly to CN V_1_ (*n* = 1), CN V_2_ (*n* = 4), or the PPF (*n =* 1) (Fig. 2). In 3 patients, distant disease spread to CNs in the orbital or periorbital area was characterized by CN thickening and enhancement, and there was no orbit or maxillofacial mass (Fig. 3).

**Table 2:** Lymphoma type, sites of primary disease, intraorbital disease appearance, and sites of neurolymphomatosis involvement in patients with neurolymphomatosis in the orbit associated with lymphoma

Patient	Lymphoma type	Site(s) of primary disease	orbital /periorbital disease appearance	Site(s) of NL involvement^a^
1	DLBCL	Maxillary sinus	Spread to V_2_	V_2_, PPF
2	DLBCL	Face	Spread to V_2_	V_2_, PPF
3	DLBCL	Masticator space, neck	Spread to PPF	V_2_, V_3_, ***PPF*,** ***CS*,** ***MC***
4	DLBCL	Face	Spread to V_2_	V_2_
5	DLBCL	Orbital/	Mass involving lacrimal gland, face, and lateral orbit	V_2_, PPF, VC
		face		
6	DLBCL	Maxillary sinus/	Maxillary sinus mass with spread to orbit	V_2_, PPF, VC
		orbit		
7	DLBCL	Orbit, neck, and chest wall	Orbital mass	V_2_, PPF
8	DLBCL	Forehead/eyebrow	Spread to V_1_	V_1_
9	DLBCL	Face	Spread to V_2_	V_2_, PPF, CS, MC
10	Marginal zone	Orbit	Lacrimal gland and posterior orbital mass	II, V_1_, V_2_, PPF, CS, MC
11	Marginal zone	Orbit, skull base	Infiltrative disease involving orbit and extraocular muscles	***II***, V_1_, ***V***_***2***_, ***PPF***, ***CS***, VC, MC
12	Marginal zone	Orbit	mass posterior orbit	II, V_2_, V_3_, PPF, CS
13	Low-grade B-cell	Masticator space, sphenoid bone, orbit	Masticator mass with spread to orbit	V_2_, PPF, CS
14	Low-grade B-cell	Orbit, nasopharynx, neck	Posterior orbital mass	V_1_, V_2_, V_3_, PPF, CS, VC
15	Mantle cell	Neck and chest	CN involvement	***II*,** ***V***_***2***_**,** ***PPF***
16	Mantle cell	Chest node	CN involvement	V_2_, ***V***_***3***_, PPF, ***MC***
17	CLL/SLL	Abdomen	CN involvement	V_1_, V_2_, PPF, CS
18	NK/T-cell	Orbit, face, nasal cavity, nasopharynx	Infiltrative disease involving nasal cavity and orbit	V_2_, V_3_, VII, PPF**,** ***CS, MC***

^a^Bold italic font denotes bilateral involvement

*CLL/SLL* chronic lymphocytic leukemia/small lymphocytic lymphoma, *CN* cranial nerve, *CS* cavernous sinus, *DLBCL* diffuse large B-cell lymphoma, *MC* Meckel’s cave, *NK* ntural killer, *PPF* pterygopalatine fossa, *VC* vidian canal

Overall, 81 sites of neurolymphomatosis were localized on MRI. The most common sites of neurolymphomatosis were V_2_ including at the infraorbital fissure or foramen rotundum (17 patients; 19 nerves), the PPF (16 patients; 19 nerves), and the cavernous sinus (9 patients; 12 nerves). CN II was involved in 4 cases and in all 4 of these cases there was also neurolymphomatosis on V2. In patient #18 there was spread of disease to involve CN VII. There was no involvement of CN I, CN III, CN IV, CN VI, or CN VIII through CN XII.

The 9 cases of DLBCL were associated with 26 sites of neurolymphomatosis, while the 9 cases of other types of lymphoma were associated with 55 sites of neurolymphomatosis. The number of sites of neurolymphomatosis was significantly lower for DLBCL than for the other types of lymphoma (*p* = 0.007). The appearance of disease in the orbits and sites of neurolymphomatosis by tumor type are described in Table 2.

### Treatment and follow-up

Sixteen patients were treated with chemotherapy and 2 with radiation (Table 1). Six of the 18 patients (33%) died during follow-up. Five of these patients had DLBCL, and 1 had low-grade B-cell lymphoma. No correlation was noted between number of sites of neurolymphomatosis and survival (*p* = 0.26).

## Discussion

Our results provide important information about the primary disease sites and patterns of spread of neurolymphomatosis in the orbit and retro-orbital cranial nerves in patients with lymphoma. Neurolymphomatosis most commonly occurred via direct spread from a solid mass in the orbit or maxillofacial region; only a few cases of neurolymphomatosis occurred via direct spread from infiltrative disease in the orbit or hematogenous spread from remote sites. CN V was affected in all cases, and the maxillary division was the division most commonly affected; CN V involvement extended to the PPF and cavernous sinus in the majority of cases. We also found that while DLBCL is the histologic type of lymphoma most often associated with neurolymphomatosis, it appears to involve fewer sites of neurolymphomatosis than other types of lymphoma and that there is no correlation between number of sites of neurolymphomatosis and survival. The mean interval between the pathologic diagnosis and MRI documentation of the full extent of neurolymphomatosis was 39 days after pathologic diagnosis.

Whereas we found that 13 of 18 patients with neurolymphomatosis in the orbit had a primary tumor in the form of a solid mass in the orbit and/or maxillofacial region, previous reports have stated that lymphoma involving the orbit appears as a well-circumscribed mass in only approximately half of cases and is diffuse in the remaining cases [9, 10]. Differences between these reports and ours may be related to patient selection: we selected patients who had a pathologic diagnosis of lymphoma and MRI evidence of neurolymphomatosis involving CN II, III, IV, V_1_, or V_2_, and this included not only patients with primary tumors that arose in the orbit but also patients with invasion of the orbit from primary tumors in locations such as the sinonasal cavity and masticator space.

Four of the patients in our study had neurolymphomatosis involving CN II, specifically the optic nerve sheath. Of these 4 patients, 2 had an associated unilateral solid mass in the posterior orbit. Both of the other 2 patients who had neurolymphomatosis involving CN II without a defined mass had bilateral lesions, and 1 had additional infiltrative disease involving the extraocular muscles. Kim et al. [2] noted that 1.3 to 12% of all lymphomas spread only to the optic nerve. In our study, the patients with CN II involvement also had spread along CN V. Other studies have shown that lymphoma may present not only with optic nerve sheath involvement but also with enlargement of the optic nerve [11, 12].

In our study, all 18 cases of neurolymphomatosis involved at least 1 of the 3 divisions of the trigeminal nerve. Lymphomas arising in the premaxillary region may spread to V_2_ via the infraorbital foramen, lymphomas arising in the maxillary sinus may spread to V_2_ via the infraorbital canal and infraorbital nerve, and lymphomas arising in the orbit may spread to V_2_ through the inferior orbital fissure. As the trigeminal nerve is a pathway for disease spread to the ocular adnexa or away from the ocular adnexa to the PPF, foramen rotundum, superior and inferior orbital fissures, cavernous sinus, Meckel’s cave, and foramen ovale, these sites are all commonly grouped together as sites likely to be affected by neurolymphomatosis along CN V [2, 13–17]. This direct communication explains the involvement of the PPF and cavernous sinus in our study.

We also found that CN V_1_ was involved in only 4 cases while V_2_ was involved in 17 cases. In our experience, both neurolymphomatosis and PNS from other tumors is more challenging to detect with MRI on V_1_ than on V_2_. This may be related to the fact that V_1_ courses through the superior orbit and is in close proximity to the superior rectus/levator palpebrae complex and may be difficult to visualize on MRI.. Another potential explanation for the more common involvement of V_2_ than of V_1_ in our study is the finding, demonstrated by multiple authors [18–20], that LMD occurs more commonly in dependent portions of the brain. This possibility could be the topic of further investigation.

In our study, only 1 CN was involved in 13 of the 18 patients (72%). We found no cases of neurolymphomatosis involving CN I, III, IV, VI, or VIII-XII. Baehring et al. [4] stated that approximately 20% of patients with neurolymphomatosis will have an isolated cranial neuropathy early in the disease course, often involving CN VII and less frequently involving CN II, IV, or V. With the exception that we found no cases of isolated neurolymphomatosis involving CN IV, which in our experience is also challenging to evaluate on MRI for neurolymphomatosis or LMD, due to its small size and location, our results were in line with what Baehring et al. stated, as only CN II, CN V, and CN VII were involved. In 4 patients there was also spread from the PPF to the vidian canal but not posteriorly to involve the greater petrosal or facial nerves. The involvement of so few cranial nerves may also be related to our selection criteria; we aimed to evaluate neurolymphomatosis in the orbit and periorbital area rather than addressing lymphoma arising in other locations. However, our findings are similar to other reports that diffuse CN involvement with neurolymphomatosis is not a common presentation [21, 22].

CN VII was directly involved in only 1 patient in our study, a patient with NK/T-cell lymphoma who also had involvement of CN V with direct spread of neurolymphomatosis via the greater petrosal nerve to the facial nerve. This pattern of CN spread is similar to the pattern reported by Cruz et al. [23], who found CN VII/VIII involvement in 1 of 6 patients with NK/T-cell lymphoma and CN V disease in 4 of the 6 patients.

In our study, half of the patients had DLBCL, and DLBCL affected fewer sites with neurolymphomatosis than other lymphoma types did. Given that DLBCL only accounts for only about 10–15% of all cases of orbital lymphoma according to most series, it was disproportionately associated with the presence of neurolymphomatosis in our series [24]. Similarly, Azevedo et al. [5] reported that involvement of multiple CNs is rare in cases of DLBCL and Stacy et al. [25] found that 57% of orbital DLBCL cases were restricted to the ocular adnexa. In contrast, marginal zone lymphoma which accounts for at least 60% of all cases of orbital lymphoma was seen in only 3 patients with neurolymphomatosis in our series [26]. We found that whereas DLBCL had a localized presentation, marginal zone, mantle cell, and NK/T-cell lymphomas involved a significantly greater number of sites of neurolymphomatosis (*p* = 0.007). In addition, the youngest patient in our study group was 24 years old suggesting that neurolymphomatosis is even rarer in the pediatric population.

In our study, 5 of the 9 patients (56%) with DLBCL died during follow-up even though DLBCL was associated with significantly fewer sites of neurolymphomatosis. However, no significant correlation was found between the number of sites of neurolymphomatosis and survival. In a multicenter review, Olsen et al. [26] studied disease-specific survival in 797 patients with orbital lymphoma. They found that the histologic subtype was the main predictor of outcome, with DLBCL and mantle cell lymphoma having the lowest 10-year disease-specific survival rates, of 41 and 32%, respectively.

Limitations of our study include the selection criteria, retrospective nature, and relatively small number of patients. It is known that perineural disease spread can be challenging to detect de novo, and our study could only include disease that had been detected radiologically. For example, patient 3# had a facial nerve palsy and potentially CN VII disease could not be detected radiographically. However, our study provides more information about neurolymphomatosis, which is a rare manifestation of lymphoma, and may serve as the basis for further investigation.

## Conclusions

Neurolymphomatosis is a rare manifestation of lymphoma that could easily be overlooked by ophthalmologists, oculoplastic surgeons, oncologists, and radiologists caring for patients with lymphoma. Based on our study results, lymphoma with neurolymphomatosis appears to arise as a solid mass in the orbit and/or maxillofacial region or via hematogenous spread to the CNs and may occur months to years after the original diagnosis of lymphoma. It is most often encountered with DLBCL and is less often seen with the typical forms of orbital lymphoma that are low-grade. The most common presentation of neurolymphomatosis in patients with lymphoma appears to be involvement of the trigeminal nerve, specifically the maxillary division including at the infraorbital fissure or foramen rotundum (V_2_), with spread further posteriorly to the PPF and cavernous sinus. Therefore, a systematic search for clinical and radiographic evidence of neurolymphomatosis is imperative for early detection. This may be especially true in patients with DLBCL.

## Data Availability

All data generated or analysed during this study are included in this published article.

## Abbreviations

CNs: Cranial nerves
DLBCL: Diffuse large B-cell
NHL: Non-Hodgkin lymphoma
NK: Natural killer
PNS: Perineural spread
LMD: Leptomeningeal disease
PPF: Pterygopalatine fossa
